# Predictive role of a novel nutrition and inflammation-based model in the short-term clinical outcomes and health-related quality of life of patients undergoing radical gastrectomy: a retrospective cohort study

**DOI:** 10.1007/s12672-025-03668-9

**Published:** 2025-10-14

**Authors:** Pengfei Li, Chunhua Zhou

**Affiliations:** 1 Department of Gastrointestinal and Anal Surgery, Chengbei District of Hangzhou First People’s Hospital (Hangzhou Geriatric Hospital), Hangzhou, 310000 China; 2 Department of Gastrointestinal and Anal Surgery, Hangzhou First People’s Hospital, Hangzhou, 310000 China

**Keywords:** Gastric cancer, Radical gastrectomy, Clinical outcomes, Nutrition, Inflammation, Health-related quality of life

## Abstract

**Objective:**

To explore the predictive value of nutritional and inflammatory indicators in the short-term clinical outcomes and health-related quality of life (HRQoL) of patients undergoing radical gastrectomy.

**Methods:**

A total of 360 patients who underwent radical resection for gastric cancer in our hospital from May 2021 to May 2023 were selected as study subjects. According to the short-term clinical outcomes recorded in the medical record system, patients were divided into the good outcome group and the poor outcome group. The general demographic data, nutritional indicators, inflammatory indicators and HRQoL scores of the two groups of patients were collected and compared. The difference indicators were included in the Logistic regression model to analyze the related factors for poor short-term clinical outcomes of patients undergoing radical resection for gastric cancer, and the Pearson correlation analysis method was used to analyze the relationship between nutritional indicators, inflammatory indicators and q HRQoL.

**Results:**

Among 360 patients who underwent radical resection for gastric cancer, 108 were divided into the poor outcome group and 252 into the good outcome group. The body mass index (BMI), prognostic nutritional index (PNI), peripheral blood neutrophil to lymphocyte ratio (NLR), platelet lymphocyte ratio (PLR), systemic immune-inflammation index (SII) and HRQoL score between the two groups were statistically significant (*P* <0.05). Logistic regression analysis showed that BMI, PNI, NLR, PLR, and SII were independent risk factors for poor short-term clinical outcomes. A prediction model including the above five predictors was established based on Logistic regression. The net benefit rate of the prediction model in the threshold range was high, indicating that the prediction model had good accuracy. Pearson correlation analysis showed that BMI, PNI, NLR, PLR, and SII were positively correlated with HRQoL score (*P* <0.05).

**Conclusion:**

BMI, PNI, NLR, PLR, and SII are important independent risk factors for poor short-term clinical outcomes in patients undergoing radical gastrectomy, and are closely related to the patients’ HRQoL. The construction of a prognostic prediction model can effectively screen high-risk groups and provide a theoretical basis for clinical intervention measures.

## Introduction

Gastric cancer is a type of stomach cancer that originates from the epithelial cells of the gastric mucosa. Patients usually have no significant symptoms in the early clinical stages, but as the disease progresses, patients will experience symptoms such as anemia, anorexia, irregular pain, and hematemesis, which seriously endanger the patient’s health and life safety [1–3]. According to Ren et al. [4], gastric cancer ranks fifth in incidence and third in mortality among malignant tumors worldwide. Its incidence and mortality rates are high worldwide. Therefore, adopting scientific and effective treatment methods is of great clinical significance to patients. In recent years, with the continuous development of science and technology, the treatment methods for gastric cancer patients have been continuously updated, but surgical treatment is still one of its main clinical treatment methods. Radical gastrectomy can effectively remove the patient’s lesions and improve the patient’s clinical symptoms, so as to achieve the effect of improving clinical outcomes and health-related quality of life (HRQoL) [5]. However, Jung et al. [6] found that patients with gastric cancer have no obvious symptoms in the early stages, and the disease usually progresses to the middle and late stages when it is discovered. Some patients have poor clinical outcomes and low HRQoL due to rapid disease progression. Therefore, finding key indicators for evaluating the short-term clinical outcomes of gastric cancer patients can be beneficial to the selection of clinical treatment options.

In recent years, tumor biology has been developing continuously, and the relationship between the occurrence and development of tumors and inflammatory response and nutritional status has gradually attracted the attention of clinical scholars [7]. Data showed that systemic inflammatory response participates in the occurrence and development of tumors through various mechanisms, including promoting the proliferation of tumor cells and inhibiting the function of the immune system [8, 9]. Zheng et al. [10] found that inflammatory markers such as neutrophil to lymphocyte ratio (NLR) and platelet to lymphocyte ratio (PLR) can effectively reflect the patient’s immune status and are closely related to the clinical outcomes of malignant tumor patients. Liu et al. [11] found that there is a close relationship between nutritional status and tumor patients. Due to the special anatomical structure of the body, gastric cancer patients are very prone to malnutrition. When the body is in poor nutritional status, it will cause the body’s immune function to decrease, reduce resistance to tumors, and increase the tumor growth rate. Additionally, the stress response of surgery will quickly deplete the body’s nutritional reserves, thereby affecting the recovery of gastric function and wound healing. Malnutrition will further increase the patient’s hospitalization time and the occurrence of complications, affecting clinical treatment effects and HRQoL.

Therefore, inflammatory and nutritional indicators play a crucial role in the short-term clinical outcomes and HRQoL of patients undergoing radical gastrectomy. Improving these indicators may help enhance patients’ HRQoL and clinical outcomes. However, few clinical studies have investigated the relationship between inflammatory and nutritional indicators and the clinical outcomes and HRQoL of patients undergoing radical gastrectomy. In-depth exploration of this topic could further predict patients’ short-term clinical outcomes and HRQoL, providing a theoretical basis for developing future treatment strategies. Based on this, this study will explore the correlation between inflammatory and nutritional indicators and the short-term clinical outcomes and HRQoL of patients undergoing radical gastrectomy. It aims to examine the potential applications of these indicators in gastric cancer treatment, offering new perspectives and strategies for clinical prevention and treatment with significant theoretical and practical implications.

## Materials and methods

### Study subjects

A total of 360 patients who underwent radical gastrectomy in our hospital from May 2021 to May 2023 were retrospectively selected as research subjects. This study has been approved by the ethics committee of our hospital, and all procedures were carried out in accordance with the ethical standards of the 1964 Declaration of Helsinki and its subsequent amendments.

### Inclusion and exclusion standards

Inclusion criteria: (1) patients who underwent elective (non-emergency, with preoperative preparation ≥ 72 h) radical resection surgery; (2) aged ≥ 18 years; (3) patients who met the relevant diagnostic criteria for gastric cancer in the National Comprehensive Cancer Network (NCCN) Clinical Practice Guidelines for Gastric Cancer [12] and were confirmed by pathological histological examination; (4) patients with complete clinical data; (5) patients who had not received preoperative neoadjuvant therapy such as radiotherapy, chemotherapy or immunotherapy. and (6) Patients undergoing laparoscopic or open surgery, including radical total gastrectomy, radical distal gastrectomy, or radical proximal gastrectomy.

Exclusion criteria: (1) patients with malignant tumors of other systems; (2) patients with severe organ dysfunction such as heart, liver, or kidney; (3) patients with systemic infection requiring systemic anti-infection treatment within 4 weeks before surgery; (4) patients with severe neurological diseases that affect HRQoL assessment (e.g., Mini-Mental State Examination (MMSE) score < 18) or cognitive dysfunction (e.g., Patient Health Questionnaire-9 (PHQ-9) score ≥ 15).

## Methods

### Data collection

This was a retrospective study. All patient data were collected through the medical record system, including age, gender, smoking history (smoking >1 cigarette/day for >1 year or quitting < 1 year), drinking history (drinking >1 unit/day for >1 year or quitting < 1 year; 1 unit = 45mL spirits/360mL beer/120mL wine), diabetes (diabetes-related diagnostic criteria in the Application of the Chinese Expert Consensus on Diabetes Classification in clinical practice [13]), hypertension (meeting the diagnostic criteria for hypertension in The Japanese Society of Hypertension Guidelines for Self-monitoring of Blood Pressure at Home (Second Edition) [14], hyperlipidemia (meeting the diagnostic criteria for hyperlipidemia in the Report of the Japan Atherosclerosis Society (JAS) Guideline for Diagnosis and Treatment of Hyperlipidemia in Japanese adults [15]), tumor location, tumor size, and histological grade.

### Nutritional indicators

The height and weight of the patients were measured, and the body mass index (BMI) of the patients was calculated by the formula (weight/height)^2^. 5 ml of fasting venous blood was collected from the patients in the morning, and the blood serum was separated by centrifugation at 3000 r/min for 10 min using a centrifuge (model: LL900, manufacturer: Luoyang Hongshi Machinery Equipment Co., Ltd.), and the samples were stored at − 80 °C. The serum albumin and peripheral blood lymphocyte count of the patients were tested and compared using a fully automatic blood biochemical analyzer (model: Boke BK-200, manufacturer: Shandong Boke Biological Industry Co., Ltd.), and the prognostic nutritional index was calculated (PNI = serum albumin + 5 × peripheral lymphocyte count).

### Inflammatory indicators

Peripheral blood samples were analyzed using the enzyme-linked immunosorbent assay (ELISA) with a double-antibody sandwich method, following the manufacturer’s protocol. Platelets, neutrophils, and lymphocyte counts were measured, and NLR, PLR, and systemic immune -inflammation index (SII) (SII=platelet count×neutrophil count/lymphocyte count) were calculated.

### HRQoL score

The HRQoL was assessed based on the Short Form 36 (SF-36) [16], which includes eight dimensions: general health, vitality, physical function, pain, physical role, emotional role, social function, and mental health. The total score is 145, with a score of < 72 indicating poor HRQoL, 72–117 indicating moderate HRQoL, and >117 indicating good HRQoL.

### Short-term clinical outcomes

Patients were followed up after surgery to observe their short-term clinical outcomes. Patients with complications such as anastomotic bleeding, anastomotic leakage, gastrointestinal obstruction, surgical site infection, pulmonary infection, abdominal infection, or death after surgery were considered as signs of poor short-term clinical outcomes. Conversely, patients without postoperative complications who were alive at the end of follow-up were considered to have good short-term clinical outcomes.

### Observation indicators

The short-term clinical outcomes and HRQoL of patients undergoing radical resection for gastric cancer were collected, and the clinical data of the poor outcome group and the good outcome group were compared. The related factors affecting the poor short-term clinical outcomes of patients were analyzed, and multivariate analysis was performed on the difference indicators of univariate analysis. A nomogram was constructed to analyze the predictive value of each factor, the accuracy of the nomogram was verified, and the correlation between nutritional indicators and inflammatory indicators and patients’ HRQoL was analyzed.

### Statistical analysis

SPSS 25.0 statistical software was used to analyze the data. Count data were expressed as n, and the *χ2* test was adopted. Normal distributed measurement data were expressed as ($\overline {\chi }$± s), measurement data that did not conform to normal distribution were expressed as [M (P_25_, P_75_)], and Z test was used. *P* < 0.05 was considered statistically significant. Multivariate logistic regression analysis was performed on the difference indicators. The variable weights were visualized and converted to a nomogram model using the “rms” package in R. Calibration curves and decision curves were drawn for internal validation. The model’s discrimination and calibration were verified using the Hosmer-Lemeshow test, the slope of the calibration curve, and the threshold probability range (10%−50%) in the decision curve. Pearson correlation analysis was used to analyze the relationship between nutritional indicators, inflammatory indicators and HRQoL.

## Results

### Short-term clinical outcomes of radical gastrectomy

According to the records of the medical record system, among the 360 patients who underwent radical gastrectomy for gastric cancer, 108 patients had poor short-term clinical outcomes (including 8 cases of anastomotic bleeding, 2 cases of anastomotic leakage, 33 cases of gastrointestinal obstruction, 10 cases of surgical site infection, 23 cases of pulmonary infection, 7 cases of abdominal infection, and 25 deaths), accounting for 30.00%, and they were divided into the poor outcome group. The remaining 252 patients (70.00%) had favorable clinical outcomes and were classified into the good outcome group.

### Univariate analysis of factors affecting the short-term clinical outcomes of patients undergoing radical gastrectomy

BMI and PNI were used as nutritional status indicators, while NLR, PLR, and SII were considered critical systemic inflammatory markers. Statistical comparisons revealed significant differences between the two groups in BMI, PNI, NLR, PLR, SII, and postoperative HRQoL (*x²* =10.106, 17.018, 12.801, 12.550, 13.901, 5.414, *P* < 0.05). These findings suggest that nutritional and inflammatory indicators are crucial factors influencing short-term clinical outcomes, with a clear correlation between postoperative HRQoL and short-term clinical outcomes (Tables 1 and 2).

**Table 1:** Analysis of general data of poor outcome group and good outcome group (n)

Indicator		Poor outcome group (*n* = 108)		Good outcome group (*n* = 252)		x²		*P*	
Age	< 60 years old	63		162		1.143		0.285	
	≥ 60 years old	45		90					
Gender	Male	75		149		3.424		0.064	
	Female	33		103					
Smoking history	Yes	68		143		1.205		0.272	
	No	40		109					
Drinking history	Yes	43		89		2.201		0.138	
	No	55		163					
Diabetes	Yes	13		22		0.942		0.332	
	No	95		230					
Hypertension	Yes	10		16		0.955		0.328	
	No	98		236					
Hyperlipidemia	Yes	4		7		0.219		0.640	
	No	104		245					
Tumor location	Low	67		151		0.142		0.707	
	High/Medium	41		101					
Tumor size	<5 cm	61		133		0.417		0.518	
	≥ 5 cm	47		119					
Histological grade	Highly differentiated	13		20		1.527		0.217	
	Poorly differentiated	95		232					
HRQoL score	Poor	75		142		5.414		0.020	
	Moderate/good	33		110					

**Table 2:** Analysis of nutrition and inflammatory indicators in the poor outcome group and the good outcome group (n)

Indicator		Poor outcome group (*n* = 108)	Good outcome group (*n* = 252)	x²	*P*
BMI	<20 kg/m²	58	90	10.106	0.001
	≥ 20 kg/m²	50	162		
PNI	< 46	63	88	17.018	< 0.001
	≥ 46	45	164		
NLR	<2	43	152	12.801	< 0.001
	≥ 2	65	100		
PLR	<187	38	140	12.550	< 0.001
	≥ 187	70	112		
SII	<531	36	138	13.901	< 0.001
	≥ 531	72	114		

### Assignment of independent variables

The short-term clinical outcomes of patients undergoing radical gastrectomy was taken as the dependent variable, and BMI, PNI, NLR, PLR, and SII were assigned as independent variables (see Table 3).

**Table 3:** Independent variable assignment

Variable	Assignment table
Short-term clinical outcomes of patients undergoing radical gastrectomy for gastric cancer	0 = good outcome, 1 = poor outcome
BMI	0 = ≥ 20 kg/m², 1 = < 20 kg/m²
PNI	0 = ≥ 46, 1= <46
NLR	0= <2, 1 = ≥ 2
PLR	0= <187, 1 = ≥ 187
SII	0= <531, 1 = ≥ 531

### Multivariate logistic regression analysis of factors affecting the short-term clinical outcomes of patients undergoing radical gastrectomy

**Fig. 1:**
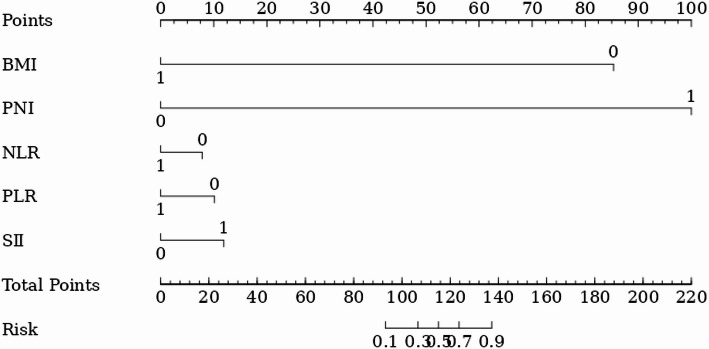
Nomogram of poor short-term clinical outcomes in patients undergoing radical gastrectomy

The stepwise forward method was used to include each factor into the logistics multivariate analysis. BMI (*OR* = 2.088, 95% *CI* = 1.321–3.300), PNI (*OR* = 2.609, 95% *CI* = 1.644–4.141), NLR (*OR* = 2.298, 95% *CI* = 1.450–3.642), PLR (*OR* = 2.303, 95% *CI* = 1.444–3.672), and SII (*OR* = 2.421, 95% *CI* = 1.512–3.877) were all independent risk factors for poor short-term clinical outcomes of patients (Table 4). According to the results of multivariate logistic regression analysis, a nomogram model was constructed for five independent risk factors: BMI, PNI, NLR, PLR, and SII. The scores corresponding to the values of each indicator were added together to obtain the total score. The scores were then converted into the predicted probability of postoperative clinical efficacy based on the nomogram (Fig. 1).

**Table 4:** Multivariate logistic regression analysis of factors affecting the short-term clinical outcomes of patients undergoing radical gastrectomy

Influencing factors	Beta value	SE	Wald X²	*P*	OR Value	OR 95% CI
BMI	0.736	0.234	9.940	0.002	2.088	1.321–3.300
PNI	0.959	0.236	16.554	< 0.001	2.609	1.644–4.141
NLR	0.832	0.235	12.533	< 0.001	2.298	1.450–3.642
PLR	0.834	0.238	12.275	< 0.001	2.303	1.444–3.672
SII	0.884	0.240	13.553	< 0.001	2.421	1.512–3.877

### Verification curve analysis

**Fig. 2:**
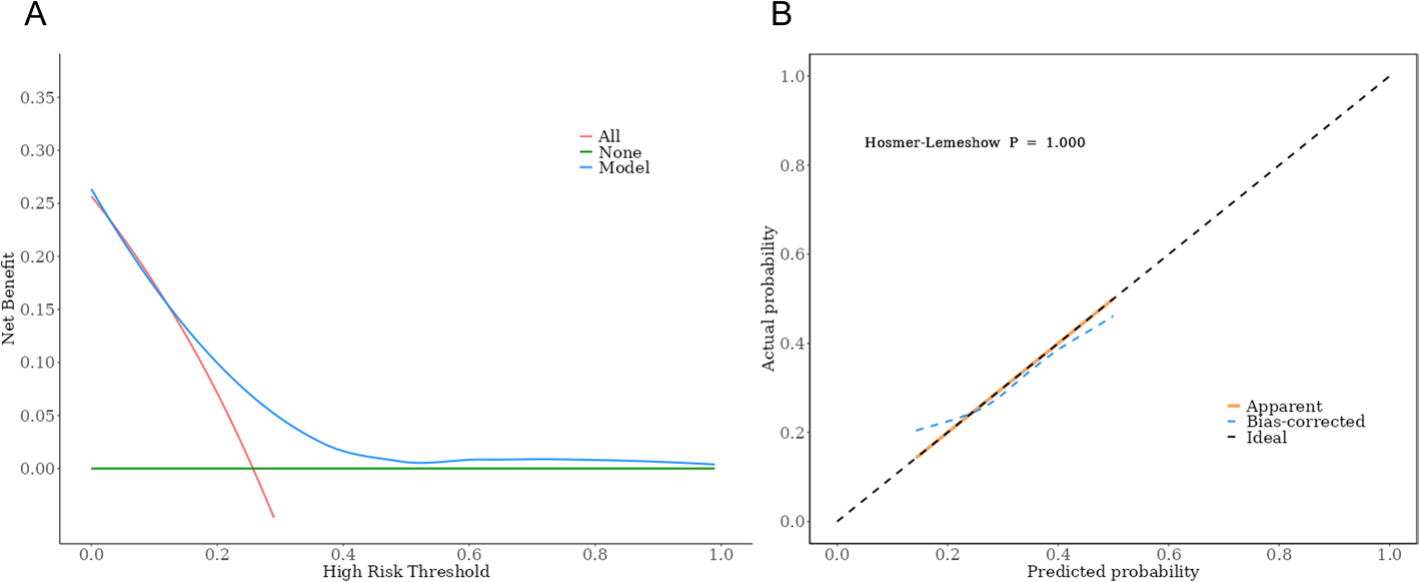
Calibration curve and decision curve

The calibration curve and reference curve of the risk prediction model showed high discrimination and calibration, and the Hosmer-Lemeshow test *P* was 1, indicating that there was no statistically significant difference between the predicted probability and the actual probability, proving that there was a high consistency between the actual risk and the predicted risk of poor short-term clinical outcomes in patients undergoing radical gastrectomy for gastric cancer. Additionally, the high net benefit rate of the prediction model proved that this model has a high clinical applicability, as shown in Fig. 2.

### Relationship between nutrition, inflammatory indicators and postoperative HRQoL

**Fig. 3:**
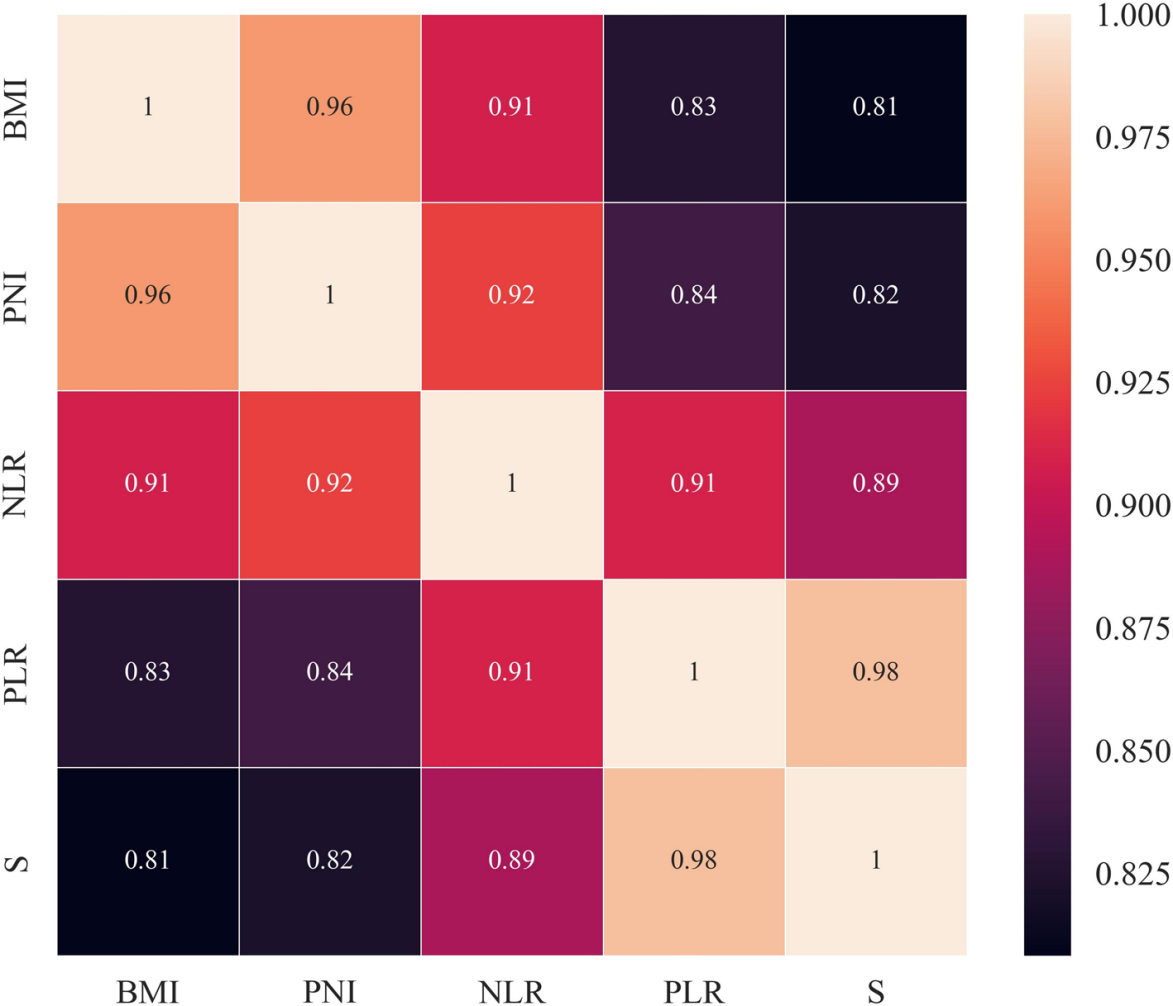
Relationship between nutrition, inflammatory indexes and postoperative HRQoL

BMI, PNI, NLR, PLR, and SII were positively correlated with postoperative HRQoL score (*r* = 0.678, 0.690, 0.747, 0.821, and 0.839, *P* < 0.05), indicating that those indicators were closely correlated with postoperative HRQoL, and the correlation was high, as shown in Fig. 3.

## Discussions

Gastric cancer is one of the most common and deadly malignancies worldwide. Although the incidence and mortality rates of gastric cancer have declined globally over the past 50 years, it remains the third leading cause of cancer-related deaths [17, 18]. Recent data indicate that there are 1.03 million new cases of gastric cancer (5.7% of all diagnosed cancers) and 783,000 deaths globally. In China, as a populous country, the incidence and mortality rates of gastric cancer remain high. According to relevant studies, surgery is currently the primary treatment for gastric cancer patients, but some still experience poor short-term clinical outcomes [19]. How to assess the surgical risk of patients before surgery, predict the short-term clinical outcomes of patients, and provide appropriate intervention measures to prolong the survival time and improve the short-term clinical outcomes of patients are the focus of clinical physicians. The present study found that among 360 patients who underwent radical resection for gastric cancer, 108 patients had poor short-term clinical outcomes, and 252 patients had good short-term clinical outcomes. The BMI, PNI, NLR, PLR, SII and postoperative HRQoL score of patients with different prognoses were statistically significant (*P* < 0.05). Logistic regression analysis showed that BMI, PNI, NLR, PLR, and SII were independent risk factors for poor short-term clinical outcomes (*OR* >1). BMI, PNI, NLR, PLR, and SII were positively correlated with postoperative HRQoL score (*P* < 0.05), indicating that the above indicators were closely related to the short-term clinical outcomes and HRQoL of patients.

Many studies have shown that BMI is a commonly used international standard for measuring the degree of fatness and health of the human body. BMI < 20 kg/m² indicates that the body may be malnourished or underweight. PNI is used to assess the patient’s preoperative nutritional status and predict the risk of postoperative complications. Both are important indicators for evaluating nutritional status [20, 21]. Triantafillidis et al. [22] stated that the preoperative nutritional status of gastric cancer patients is an important factor affecting the patient’s survival prognosis, and the patient’s nutritional status also indirectly reflects the immune function. According to relevant research reports, low nutritional levels will cause gastric cancer patients to significantly weaken their ability to destroy malignant tumor cells, increase the risk of distant metastasis in patients, and have a great negative impact on the patient’s short-term clinical outcomes. Therefore, for cancer patients, preoperative nutritional screening is of utmost importance [23]. In this study, BMI and PNI were important factors affecting the patient’s poor short-term clinical outcomes and had a certain predictive value. The reason may be that when the patient’s BMI is less than 20 kg/m² and PNI is less than 46, it indicates poor nutritional status, which leads to a decrease in the number and activity of immune cells. This increases the risk of complications, such as infection caused by surgical trauma, and reducing the clinical recovery process; and poor nutritional status will affect the wound healing speed, tissue repair ability and the body’s overall recovery ability, affect the body’s metabolism and immune function, and make tumor cells more likely to grow and spread in the body, thereby increasing the risk of poor surgical outcomes in patients, which further causes poor HRQoL for patients, This is in line with the views of Miyamoto et al. [24].

There are a large number of inflammatory cells in tumor tissues. Many clinical studies have observed that the higher the degree of inflammatory response in gastric cancer patients, the worse the prognosis. They emphasize that the inflammatory response of the body is of great clinical significance. Among them, NLR and PLR are important inflammatory markers, and SII is a new scoring system for evaluating the function of the immune system. They can reflect the patient’s inflammation and immune response [25, 26]. Rudolf Virchow first reported the relationship between inflammation and cancer in 1863. From the detection of leukocytes infiltrating in tumor tissues, he inferred that cancer-related inflammatory response is a non-specific reaction that promotes tumor proliferation, angiogenesis, tumor cell migration, invasion, anti-apoptotic signaling pathway activation and metastasis [27, 28]. Gallyas Jr et al. [29] suggested that cancer induces inflammation, which leads to the activation of transcription factors that further increase the inflammatory response, such as NF-kB, STAT3, and HIF-1a, which are regulated to produce important tumor growth-promoting cytoking. In this study, NLR, PLR, and SII were all independent risk factors for poor short-term clinical outcomes and had certain predictive value. The reason may be that the above indicators can be used to assess the activation of tumor inflammatory pathways and the body’s immune status. The derived reactive oxygen species further reduce the adhesion-promoting properties of the extracellular matrix and inhibit tumor cell apoptosis by activating NF-kB and STAT3. If these indicators rise abnormally before surgery, it indicates that a systemic inflammatory response occurs, resulting in lymphopenia, a significant decrease in the number of T4 helper lymphocytes and innate immune cells, and an increase in the number of T8 inhibitory lymphocytes. These mechanisms lead to accelerated tumor progression, invasion of surrounding tissues, and metastasis to distant tissues and organs, increasing the risk of poor surgical outcomes. The views of Wang et al. [30] further support this. Previous studies have shown that the inflammatory response itself is a defensive response of the body to a certain stimulus or injury. The presence of an inflammatory response before surgery indicates that the body is in a state of stress, consuming a large amount of energy and nutrients, resulting in reduced immune function in patients, increasing the risk of postoperative complications and the possibility of poor recovery, causing serious physiological discomfort to patients, and thus affecting the HRQoL after surgery [31].

According to the TRIPOD guidelines, the nomogram is an intuitive and effective prediction tool that plays an important role in medical research and clinical practice. It helps to improve the readability and clinical application value of the prediction model. In this study, the nomogram prediction model was constructed by combining various factors, and the complex multifactor regression equation was converted into a graph, making the abstract data outcomes visual and readable. The approximate probability of poor clinical efficacy was evaluated by different predictive variables, and the calibration curve and decision curve were drawn. The Hosmer-Lemeshow test P was 1, showing that there was no statistically significant difference between the predicted probability and the actual probability, and the net benefit rate of the prediction model within the threshold range was higher than the two extreme lines, indicating that the model predicted a higher net benefit value and had a higher predictive efficiency, which means that the prediction model can be used as an auxiliary decision-making tool. This shows that predictions based on the above factors can provide a reference for the early diagnosis of high-risk groups with poor therapeutic effects in the clinic and the implementation of relevant intervention measures.

The limitations of this study are as follows: (1) This study is based on a small sample size and single-center design. The small sample may increase the difference between sample statistics and population parameters, making the results limited in scope. Additionally, the research was conducted in a single region, which may affect the generalizability of the findings to other populations or environments. (2) The nomogram has not been externally validated, and its applicability in broader clinical settings requires further research. Overall, the predictive model is an auxiliary tool, and its practical application must be considered in the context of individual clinical conditions.

## Conclusion

In conclusion, there are multiple factors influencing the occurrence of poor short-term clinical outcomes in patients undergoing radical gastrectomy for gastric cancer, including BMI, PNI, NLR, PLR, and SII. These factors are closely related to the patients’ postoperative HRQoL. By constructing a risk prediction model and screening high-risk groups for appropriate interventions, this study provides a theoretical basis for improving clinical efficacy. In future studies, external verification will be added to improve it and further provide strong support for clinical practice.

## Data Availability

The data that support the findings of this study are available from the corresponding author, upon reasonable request.
